# The prognostic value of CT radiomic features for patients with pulmonary adenocarcinoma treated with EGFR tyrosine kinase inhibitors

**DOI:** 10.1371/journal.pone.0187500

**Published:** 2017-11-03

**Authors:** Hyungjin Kim, Chang Min Park, Bhumsuk Keam, Sang Joon Park, Miso Kim, Tae Min Kim, Dong-Wan Kim, Dae Seog Heo, Jin Mo Goo

**Affiliations:** 1 Department of Radiology, Seoul National University College of Medicine, Seoul, Korea; 2 Institute of Radiation Medicine, Seoul National University Medical Research Center, Seoul, Korea; 3 Seoul National University Cancer Research Institute, Seoul, Korea; 4 Department of Internal Medicine, Seoul National University Hospital, Seoul, Korea; Peking University People's Hospital, CHINA

## Abstract

**Purpose:**

To determine if the radiomic features on CT can predict progression-free survival (PFS) in epidermal growth factor receptor (*EGFR*) mutant adenocarcinoma patients treated with first-line EGFR tyrosine kinase inhibitors (TKIs) and to identify the incremental value of radiomic features over conventional clinical factors in PFS prediction.

**Methods:**

In this institutional review board–approved retrospective study, pretreatment contrast-enhanced CT and first follow-up CT after initiation of TKIs were analyzed in 48 patients (M:F = 23:25; median age: 61 years). Radiomic features at baseline, at 1^st^ first follow-up, and the percentage change between the two were determined. A Cox regression model was used to predict PFS with nonredundant radiomic features and clinical factors, respectively. The incremental value of radiomic features over the clinical factors in PFS prediction was also assessed by way of a concordance index.

**Results:**

Roundness (HR: 3.91; 95% CI: 1.72, 8.90; P = 0.001) and grey-level nonuniformity (HR: 3.60; 95% CI: 1.80, 7.18; P<0.001) were independent predictors of PFS. For clinical factors, patient age (HR: 2.11; 95% CI: 1.01, 4.39; P = 0.046), baseline tumor diameter (HR: 1.03; 95% CI: 1.01, 1.05; P = 0.002), and treatment response (HR: 0.46; 95% CI: 0.24, 0.87; P = 0.017) were independent predictors. The addition of radiomic features to clinical factors significantly improved predictive performance (concordance index; combined model = 0.77, clinical-only model = 0.69, P<0.001).

**Conclusions:**

Radiomic features enable PFS estimation in *EGFR* mutant adenocarcinoma patients treated with first-line EGFR TKIs. Radiomic features combined with clinical factors provide significant improvement in prognostic performance compared with using only clinical factors.

## Introduction

Lung cancer is the leading cause of cancer death worldwide and non-small cell lung cancer (NSCLC) is the largest subgroup, occupying about 85% of cases [1]. The prognosis of NSCLC still remains poor with no more than a 10-month median overall survival rate with conventional chemotherapy [2]. Several molecular-targeted agents, including epidermal growth factor receptor (EGFR) tyrosine kinase inhibitors (TKIs), have emerged in the recent decades along with the concept of personalized medicine. Large-scale clinical trials have repeatedly shown the benefits of EGFR TKI in *EGFR* mutation-positive NSCLC patients [2]. For example, the OPTIMAL study compared erlotinib with chemotherapy as a first-line treatment in Asian patients which demonstrated that EGFR TKI could significantly prolong progression-free survival (PFS) (median PFS 13.1 months versus 4.6 months) [3].

Despite their dramatic initial responses and prolonged survival, all of the patients eventually developed resistance to EGFR TKI [1]. The median PFS after treatment with a first-generation EGFR TKI in patients with *EGFR* mutations is typically less than one year [1]. Thus, prediction of PFS in these patients is significant as the predicted survival before the initiation of therapy may guide the aggressiveness of treatment, or may help to prepare for additional treatment options, at the estimated time of acquiring resistance.

Prediction of treatment responses and survival rates, based on images from patients receiving EGFR TKI, has been investigated by several researchers [4–10]. They reported the utility of quantitative parameters of positron emission tomography (PET) or computed tomography (CT) in depicting patient prognosis. Recently, radiomic approaches, which analyze the gray level of pixels and their spatial distribution with high-throughput feature extraction, have been suggested and a few studies have shown compelling evidence for the potential of this method in NSCLC patients [5, 11–15]. However, the prognostic implication of CT radiomic features in a homogeneous set of patients with adenocarcinoma and *EGFR*-sensitizing mutations has yet to be described.

Therefore, we aimed to determine if the radiomic features on CT images at baseline and first follow-up, or the percentage change between baseline and first follow-up, can predict PFS in *EGFR*-mutant adenocarcinoma patients treated with first-line EGFR TKIs. We also sought to identify the incremental value of radiomic features over conventional clinical factors in PFS estimation.

## Materials and methods

This retrospective analysis was approved by the institutional review board of Seoul National University Hospital (IRB No. 1609-060-791) with waivers of informed consent from involved patients as the data were analyzed retrospectively and anonymously.

### Study population

We retrospectively reviewed a consecutive database of NSCLC patients and identified 415 patients who were treated with first-line EGFR TKIs (gefitinib or erlotinib) until disease progression from October 2005 to April 2015 [10, 16–19]. Among these patients, the study population was determined based on the following criteria: patients with 1) biopsy-confirmed *EGFR*-mutant adenocarcinoma, 2) at least one measurable lung lesion (≥10mm), 3) contrast-enhanced chest CT scans taken prior to (baseline) and after EGFR TKIs (first follow-up), 4) CT scans reconstructed with filtered back projection, and 5) CT scans of 2.5–3mm slice thickness (the routine follow-up CT protocol of our institution is comprised of 2.5 or 3mm thicknesses). CT scans reconstructed with iterative reconstruction algorithms were excluded as iterative reconstruction may increase the variability of radiomic features [20]. Patients were excluded if they had lesions with adjacent atelectasis or consolidation which could impede proper tumor segmentation.

**Fig 1:**
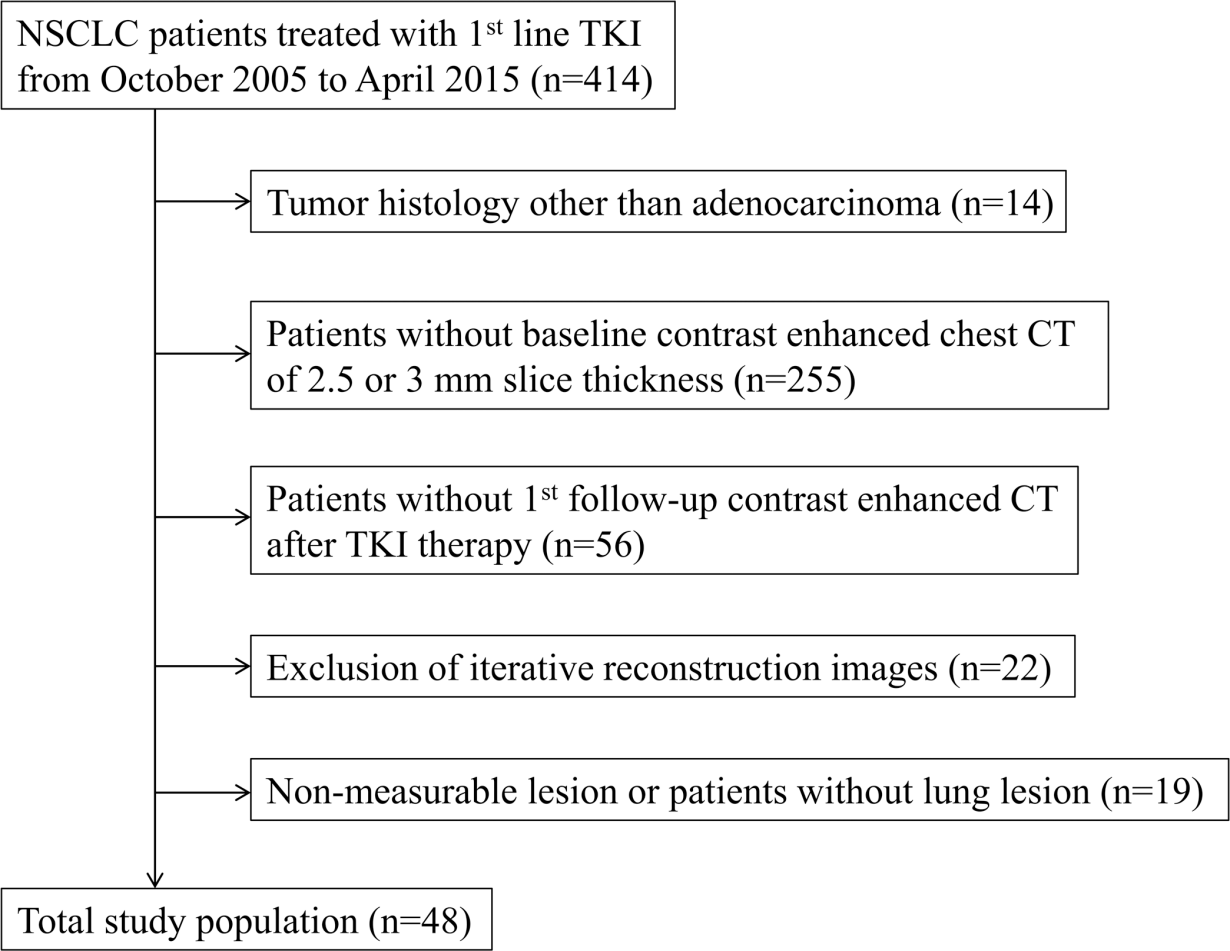
Flow chart of patient selection including inclusion, and exclusion, criteria.

The inclusion and exclusion criteria yielded 48 patients for analysis (Fig 1). There were 23 men (median age, 65 years; range, 42–85 years) and 25 women (median age, 61 years; range, 38–85 years). 46 patients were treated with gefitinib and two patients received erlotinib. The median interval between the baseline (before TKI initiation) and first follow-up CT was 9.6 weeks (interquartile range, 7.9–11.0 weeks). Detailed patient characteristics, including clinical prognostic factors and TKI treatments, are described in Table 1.

**Table 1:** Patient characteristics, clinical factors, and TKI treatment.

Characteristics	Category	Value
Median age (year)		61 (38–85)
Sex	Male	23 (47.9)
	Female	25 (52.1)
Stage	IIIB	2 (4.2)
	IV	46 (95.8)
Smoking status	Current or ex-smoker	22 (45.8)
	Never smoker	26 (54.2)
ECOG PS^a^	0	1 (2.1)
	1	33 (68.8)
	2	2 (4.2)
Extrathoracic metastasis	Absent	17 (35.4)
	Present	31 (64.6)
Tumor diameter at baseline (mm)^b^		30.5 (10.0–78.2)
Tumor diameter at first follow-up (mm)^b^		22.4 (6.2–64.6)
Sensitizing *EGFR* mutation	Exon 18 G719	1 (2.1)
	Exon 19 deletion	18 (37.5)
	Exon 21 L858R	29 (60.4)
EGFR TKI	Gefitinib	46 (95.8)
	Erlotinib	2 (4.2)
Treatment response at first follow-up	Responder	25 (52.1)
	Non-responder	23 (47.9)
Progression-free survival (month)^c^		9.7 (5.0–13.8)

Note: Unless otherwise specified, data are numbers of patients (with percentages in parentheses).

^a^Data were not available in 12 patients.

^b^Data are median (with range of data in parentheses).

^c^Data are median (with interquartile range in parentheses).

ECOG PS, Eastern Cooperative Oncology Group Performance Status Score; *EGFR*, epidermal growth factor receptor; TKI, tyrosine kinase inhibitors

### CT image acquisition

CT scans were performed with six different scanners from three manufacturers. All patients underwent contrast-enhanced CT scans from lung apex to base at suspended maximum inspiration. Detailed information regarding the scanning protocols can be found in S1 Protocol.

In addition, a separate CT dataset of solid pulmonary nodule from 43 patients was prepared to analyze inter-reader intraclass correlation coefficients (ICCs) of radiomic features. CT examinations were performed using a 64-detector row Definition scanner at full inspiration state. Detailed scanning parameters are also described in S1 Protocol.

### Clinical factors and PFS

Patient age, sex, stage (according to the 7^th^ edition of *The American Joint Committee on Cancer Staging Manual*), smoking status, Eastern Cooperative Oncology Group (ECOG) Performance Status Score (PS), presence of extrathoracic metastasis, and *EGFR* sensitizing mutation were recorded from electronic medical records. Baseline tumor size, before EGFR TKI initiation and tumor size at first follow-up were also obtained. Tumor size (longest diameter) was measured on an axial plane of CT image using electronic caliper. In addition, treatment response of patients assessed at first follow-up CT was also recorded. Patients were classified into either responders (complete or partial remission) or nonresponders (stable or progressive disease) based on Response Evaluation Criteria in Solid Tumors (RECIST) version 1.1 criteria [21]. Lastly, PFS was measured from the date of EGFR TKI therapy initiation until the date of progression (or any cause of death).

### Radiomic feature extraction

Nodule segmentation was processed as follows: First, digital imaging and communications in medicine (DICOM) files were transferred from the picture archiving and communication system (PACS) to a personal computer and then loaded to an in-house software program (Medical Imaging Solution for Segmentation and Texture Analysis) [22–26]. This in-house software program was implemented using dedicated C++ language with Microsoft Foundation Classes (Microsoft, Redmond, WA). The tumor boundary was segmented manually with freehand drawing on each axial slice of CT images to include the entire tumor volume. Segmentation was performed for a dominant measurable lung lesion (one lesion per patient).

After nodule segmentation, radiomic features were extracted automatically from the software program. We obtained a total of 37 features. The features types were: 1) first-order statistics based features (15 features), 2) size and shape features (8 features), 3) gray-level co-occurrence matrix (GLCM) based features (5 features), 4) gray-level run-length matrix (GLRL) based feature (1 feature), and 5) wavelet transformed GLRL features (8 features) (Table 2).

**Table 2:** Extracted radiomic features.

Group	Feature	No.
First-order statistics	mean, standard deviation, variance, skewness, kurtosis, entropy, homogeneity, energy, C5, C10, C25, C50, C75, C90, C95	15
Size and shape	volume, effective diameter, surface area, sphericity, discrete compactness, shape compactness 1, shape compactness 2, roundness	8
GLCM	moments, angular second moment, inverse difference moment, contrast, entropy	5
GLRL	Grey-level nonuniformity	1
Wavelet transformation	decompositions of grey-level nonuniformity	8

C5, C10, …, C95, percentile value of the cumulative histogram; GLCM, gray-level co-occurrence matrix; GLRL, gray-level run-length matrix

### Exclusion of unstable features

Nodule segmentation was initially performed by one radiologist (H.K. with 7 years of experience in chest CT) and one CT technician (M.L. with 5 years of research experience in chest CT) for the independent, solid-pulmonary nodule CT datatset to evaluate the inter-reader variability of radiomic features and to identify any unstable features for exclusion. ICCs were calculated based on the two sets of radiomic features from each observer. Features with inter-reader ICC <0.9 were excluded from further analysis.

After exclusion of unstable features, a radiologist (H.K.) conducted segmentation for the EGFR TKI patient CT dataset for the following analysis.

### Statistical analysis

Radiomic features were extracted from both baseline and first follow-up CT scans. Then, the percentage change of radiomic features between baseline and first follow-up was calculated as follows:

$$Percentage\;change\;of\;feature\;A=\frac{\left[baseline\;CT\;feature\;A–first\;follow-up\;CT\;feature\;A\right]}{baseline\;CT\;feature\;A}$$

Therefore, a total of three radiomic feature sets (baseline, first follow-up after TKI, and percentage change) were acquired for each patient.

With the radiomic features, the pairwise Pearson correlation coefficient was calculated and features with a correlation coefficient of >0.95 were grouped together. This procedure was performed three times with baseline CT features, first follow-up CT features, and percentage features, respectively. Based on the univariate Cox proportional hazards regression analysis, a feature with the smallest P-value was selected from each group. Dichotomized radiomic features were used as input variables to the Cox regression analysis. Dichotomization with optimal cutoff was determined, which showed the most significant split at log-rank test [27]. After dimensionality reduction, the P-values of remnant features were adjusted for multiple comparisons using the Benjamini-Hochberg method. We chose the top four features, based on the corrected P-value, to limit the possibility of over-fitting (according to the Harrell guideline that the number of observations should exceed the number of selected features by, at least, a factor of ten) [28]. These features were used as input variables into the multivariate Cox regression analysis with backward stepwise selection. Independent predictors of PFS were identified from multivariate Cox regression analysis and hazards ratio (HR) and a 95% confidence interval (CI) was determined. Concordance index and its 95% CI were calculated.

Along with the radiomic model for PFS prediction, we investigated the discriminative power of conventional clinical factors for risk stratification in advanced adenocarcinoma patients. ECOG PS was not included in the regression analysis as it was missing in 12 patients. Tumor stage was also excluded (as most patients (95.8%) were stage IV). Age was dichotomized with optimal cutoff. With variables exhibited a P-value of <0.20 at univariate analysis, a multivariate Cox regression analysis was conducted with backward stepwise selection.

Finally, we aimed to compare the incremental value of radiomic features against the conventional clinical factors. Therefore, we built a combined model incorporating both radiomic features and clinical factors. We assessed the performance of the model with concordance index and compared it with the radiomic model and clinical-factor model. Comparison of concordance indices was assessed by means of bootstrapping, with 1,000 repetitions and paired t-tests, performed among values.

Statistical analyses were performed using SPSS 19.0 (IBM SPSS Statistics, Armonk, NY, USA) and R software, version 3.2.2 (http://www.R-project.org). Survival analyses were performed with survival package and bootstrapping was executed with boot package in R. A P-value <0.05 was considered as statistical significance.

## Results

### Clinical characteristics and PFS

There was no significant difference in age between male and female participants (P = 0.992). The median PFS was 9.7 months (range, 0.3–29.4 months; interquartile range, 5.0–13.8 months). There were 25 (52.1%) responders and 23 (47.9%) nonresponders at first follow-up CT.

### Feature selection

Six features [kurtosis, skewness, wavelet grey-level nonuniformity HHL (high- or low-pass filter along x-, y-, and z-direction), fifth percentile of the cumulative histogram (C5), standard deviation and variance] with inter-reader ICC <0.9 (based on the independent, solid-pulmonary nodule CT dataset) were removed. Then, a pairwise Pearson correlation coefficient was calculated and a univariate Cox regression analysis was performed with the remaining 31 features. Six features were further excluded from the baseline CT features (C10, C25, C50, C90, effective diameter, and wavelet grey-level nonuniformity LHL), eight features were excluded from the first follow-up CT features (mean, C25, C50, C90, volume, effective diameter, surface area, and wavelet grey-level nonuniformity LHL), and two features (volume and effective diameter) were removed from the percentage features. The P-values of the remnant features were adjusted for multiple comparisons using the Benjamini–Hochberg procedure. After correction, 19 features showed a P-value smaller than 0.01 (Table 3). Among them, 11 features were extracted from the first follow-up CT and eight features were extracted from the baseline CT.

**Table 3:** Univariate Cox regression analysis of 19 radiomic features.

Feature	Hazard ratio	Confidence interval		P-value
		Lower	Upper	
Discrete compactness_FU	4.13	2.13	7.99	0.001
Roundness_FU	5.51	2.48	12.25	0.001
GLN_FU	4.23	2.19	8.18	0.001
GLCM contrast_FU	0.27	0.14	0.52	0.001
C75_FU	3.9	1.98	7.69	0.001
Compactness1	3.86	1.93	7.72	0.002
Compantness1_FU	3.57	1.85	6.87	0.002
Volume	3.85	1.91	7.77	0.002
LLL_FU	3.27	1.7	6.29	0.003
LLL	3.93	1.82	8.5	0.004
Energy_FU	3.1	1.62	5.94	0.005
GLCM ASM	0.31	0.16	0.62	0.006
GLN	3.37	1.64	6.94	0.006
Discrete compactness	2.79	1.51	5.17	0.006
GLCM contrast	0.32	0.16	0.64	0.006
C10_FU	2.89	1.52	5.5	0.006
GLCM entropy	3.12	1.55	6.25	0.006
GLCM entropy_FU	2.85	1.5	5.41	0.006
C95_FU	3.51	1.6	7.68	0.007

Note: Features with underbar and ‘FU’ denote that those are extracted from the first follow-up CT.

ASM, angular second moment; C10, tenth percentile at cumulative histogram; C75, seventy-fifth percentile at cumulative histogram; C95, ninety-fifth percentile at cumulative histogram; GLN, grey-level nonuniformity; GLCM, gray-level co-occurrence matrix; LLL, wavelet decomposition of grey-level nonuniformity with directional filtering with low-pass filter along x-, y- and z-direction

**Fig 2:**
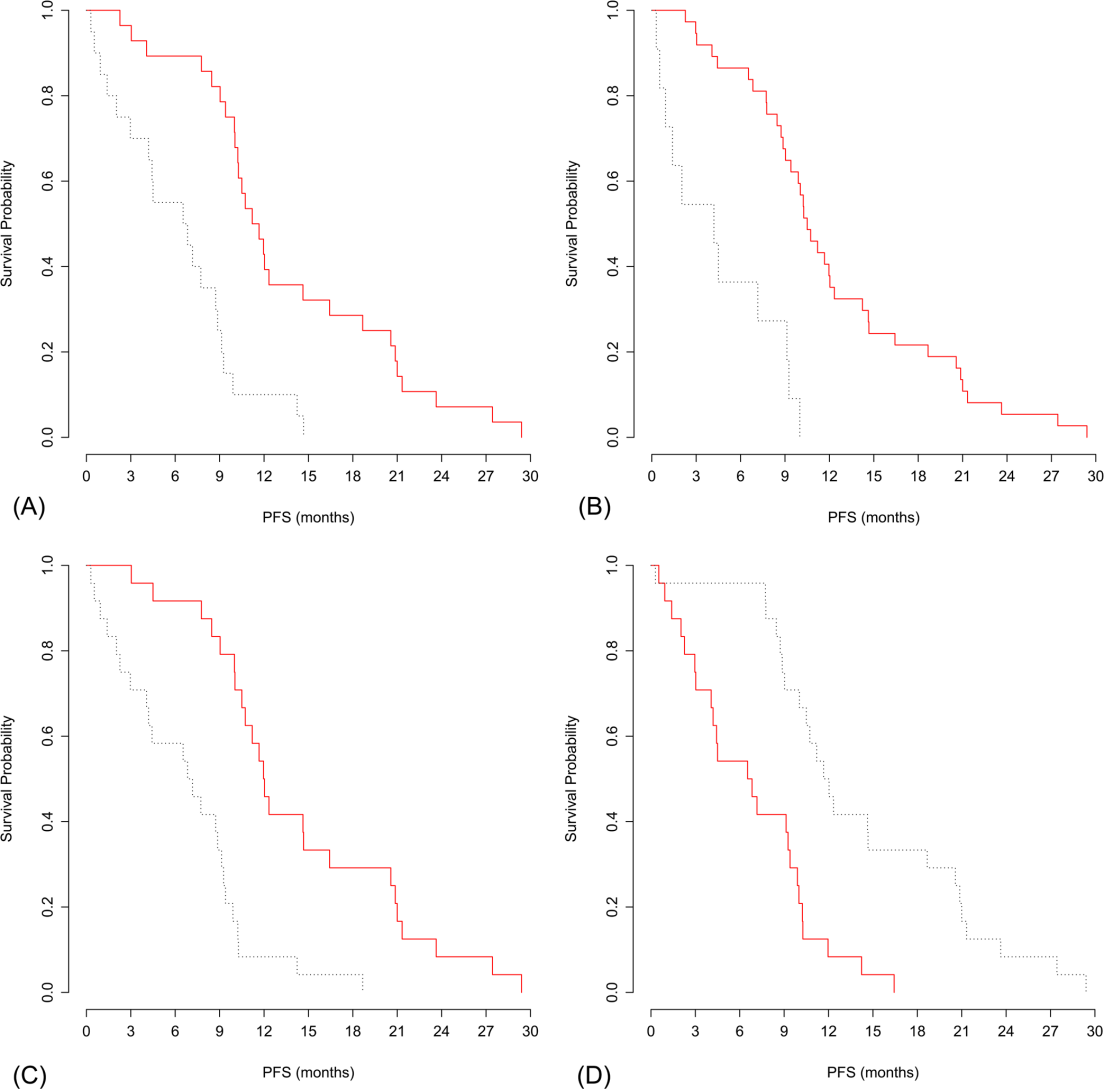
Graphs of Kaplan-Meier risk groups based on the optimal cutoff of the four radiomic features. (A) discrete compactness (cutoff: 0.0487), (B) roundness (cutoff: 0.7817), (C) grey-level nonuniformity (cutoff: 31.93), and (D) GLCM contrast (cutoff: 6066). GLCM, gray-level co-occurrence matrix.

The final top four features were discrete compactness, roundness, grey-level nonuniformity, and GLCM contrast which were all extracted from the first follow-up CT (Fig 2). The optimal cutoff values used to dichotomize these four features were 0.0487 [hazard ratio (HR): 4.13; 95% CI: 2.13, 7.99, P<0.001] for discrete compactness, 0.7817 (HR: 5.41; 95% CI: 2.45, 11.96, P<0.001) for roundness, 31.93 (HR: 4.23; 95% CI: 2.19, 8.18, P<0.001) for grey-level nonuniformity, and 6066 (HR: 0.27; 95% CI: 0.14, 0.52, P<0.001) for GLCM contrast.

### Multivariate Cox regression analysis

**Fig 3:**
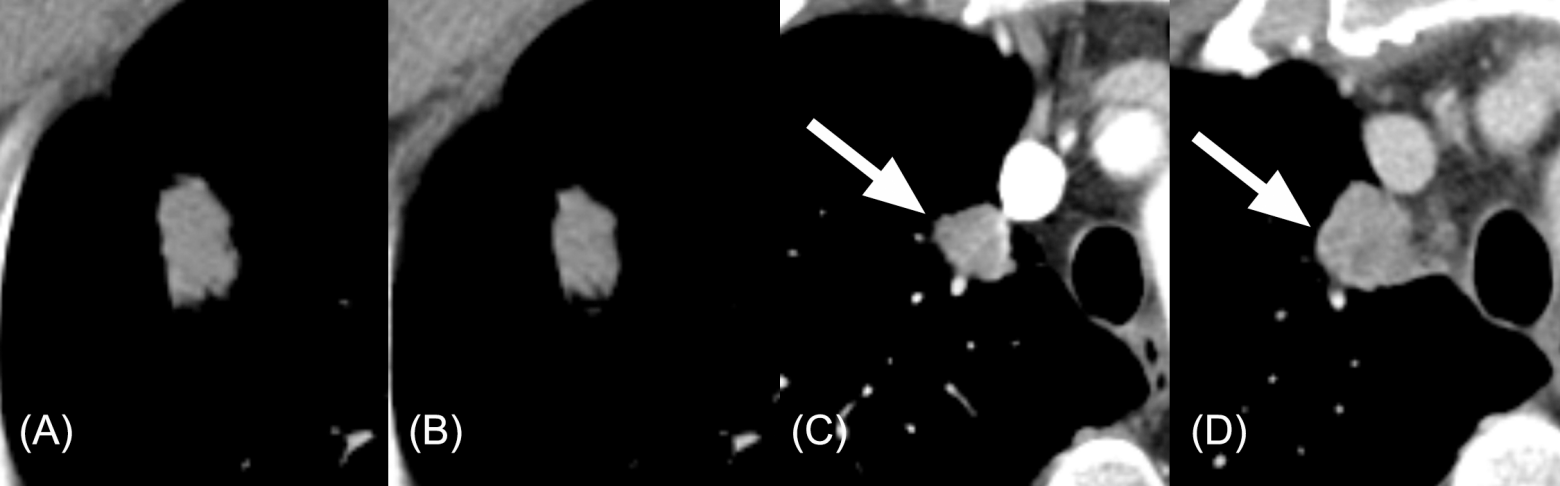
Radiomic features and their association with progression-free survival (PFS). (A) On the baseline CT, roundness of the tumor was 0.7352 and grey-level nonuniformity was 23.06. (B) On the first follow-up CT (9 weeks later), roundness was 0.7355 (percent change: 0.0422%) and grey-level nonuniformity decreased to 16.93 (percent change: -26.57%). PFS in this patient was 23.6 months. (C) For this patient, whose PFS was only 1.4 months, tumor roundness was 0.8591 and grey-level nonuniformity was 24.13 on the baseline CT. (D) On the first follow-up CT (9 weeks later), grey-level nonuniformity increased to 43.11 (percent change: 78.63%), while the roundness was 0.8040 (percent change: -6.411%).

A multivariate Cox regression analysis performed with the four features indicated that roundness (HR: 3.91; 95% CI: 1.72, 8.90; P = 0.001) and grey-level nonuniformity (HR: 3.60; 95% CI: 1.80, 7.18; P<0.001) were independent predictors of PFS (Fig 3). The concordance index of the radiomic model was 0.73 (95% CI: 0.65, 0.82).

The Cox regression analysis was also performed, with clinical factors and treatment response according to RECIST 1.1, on the first follow-up. The optimal cutoff value for age dichotomization was 70.5 (HR: 1.68; 95% CI: 0.86, 3.27, P = 0.120). A multivariate Cox regression analysis revealed that patient age (HR: 2.11; 95% CI: 1.01, 4.39; P = 0.046), baseline tumor diameter (HR: 1.03; 95% CI: 1.01, 1.05; P = 0.002), and treatment response (HR: 0.46; 95% CI: 0.24, 0.87; P = 0.017) were independent clinical predictors. The concordance index of the clinical-factors model was 0.69 (95% CI: 0.60, 0.79) and was significantly lower than that of the radiomic model (P<0.001).

**Fig 4:**
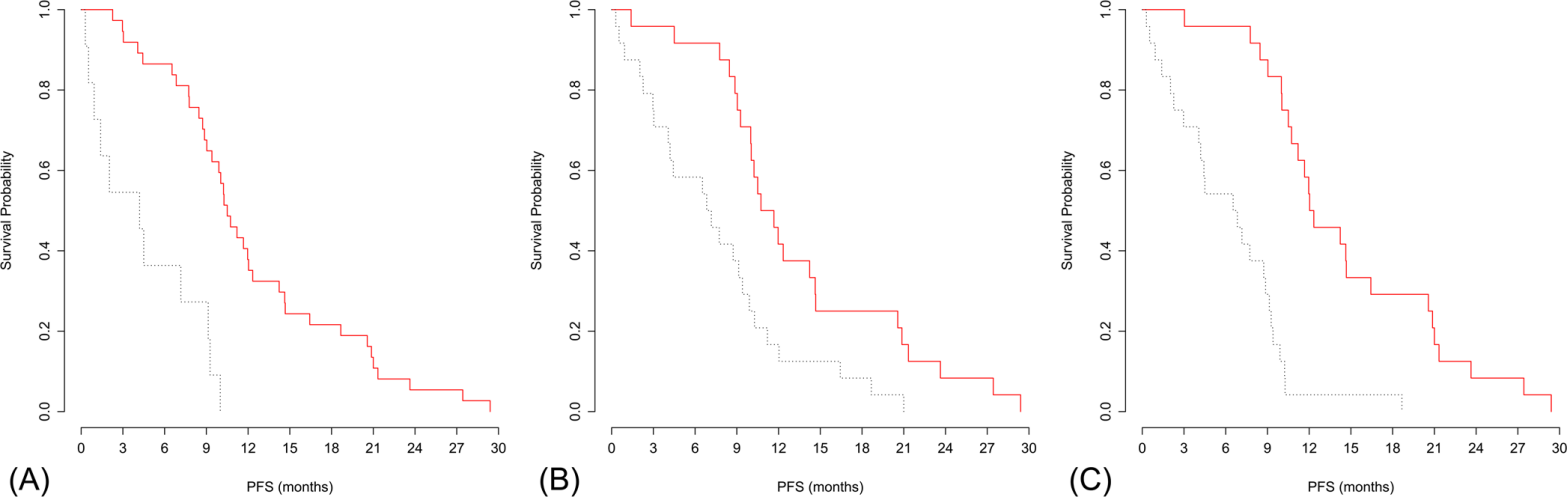
Kaplan-Meier plots demonstrating the performance of each estimation model. Patients were divided in to low- and high-probability groups for progression-free survival according to the median value of output from (A) the radiomic model (HR: 5.34, 95% CI: 2.42, 11.76; P<0.001, (B) the clinical-factor model (HR: 2.51, 95% CI: 1.37, 4.59; P = 0.003), and (C) the combined model (HR: 5.49, 95% CI: 2.77, 10.89; P<0.001). CI, confidence interval; HR, hazard ratio.

Next, we combined the radiomic features with clinical factors to create an integrated prediction model in order to evaluate the incremental value of radiomic features against conventional clinical factors. The concordance index of the combined model was 0.77 (95% CI: 0.67, 0.86), which was significantly higher than those of either the radiomic model or the clinical-factor model (both P<0.001). The addition of radiomic features to the clinical factors significantly improved the predictive performance of the regression model. Kaplan-Meier plots of the radiomic model, clinical-factor model, and combined model appear in Fig 4.

## Discussion

We have demonstrated that the incorporation of radiomic features along with conventional clinical factors was superior to the performance of clinical-factors alone model in prediction of PFS in *EGFR*-mutant adenocarcinoma patients treated with first-line EGFR TKIs. The radiomic features alone also showed a better outcome in PFS estimation than the sole employment of clinical factors.

Recently, several studies evaluated the prognostic value of radiomic features [5, 11–14, 22, 29, 30]. However, most studies included patient information, such as heterogeneous histology, stage, and treatment. Our study population consisted of patients with advanced-stage (stage IV in 46 out of 48 patients) adenocarcinoma with sensitizing *EGFR* mutations and all of them were treated with EGFR TKI as their first-line systemic therapy. The development of a model which enables risk stratification in clinically-similar patients can be very useful for optimizing treatment plans for individual patients.

It is promising that the radiomic features from the first follow-up CTs promoted PFS prediction. Early prediction of PFS may enable physicians to determine the right time to perform additional biopsies in order to identify acquired resistance such as T790M mutation for screening eligible patients for the novel agent [31]. Treatment response according to RECIST at first follow-up or baseline tumor diameter can also act as indicators in estimating patient survival [32]. However, we showed that the addition of radiomic features to the clinical factors (including treatment response) had a higher discriminative power in PFS prediction than the use of clinical factors alone.

As for the radiomic features of our study, roundness is a three-dimensional morphological index which measures the surface smoothness and structural distortion as the ratio between the diameters of enclosing, and enclosed, spheres based on surface voxels [33]. We added the additional component of radial displacement for surface smoothness. Grey-level nonuniformity is a measure of tumor heterogeneity and is calculated with GLRL [11]. It is interesting that the combination of a morphological index and a texture feature (indicating intratumoral heterogeneity), which are both beyond the perception of the human eye, can work together as a prognostic imaging biomarker. A few studies of radiomic analysis showed that the intratumoral heterogeneity on imaging yielded prognostic information in NSCLC patients [5, 11–13, 22, 30]. Aerts et al. [11] demonstrated that the performance of radiomic signatures, including intensity feature, size feature, and texture feature of grey-level nonuniformity was better than that of TNM staging. Huang et al. [12] also showed that homogeneity, kurtosis, and uniformity using a filter method were independent biomarkers for the estimation of disease-free survival in early stage (I-II) NSCLC. In a study by Fried et al. [13], disease solidity (volume metric) and GLCM energy were useful for survival prediction in patients with stage III NSCLC. The common finding in these studies, including our own, was that the incorporation of both radiomic features and clinical prognostic factors significantly improved the risk stratification of patients compared with the sole use of the clinical-factors model.

With regard to the NSCLC patients receiving EGFR TKI, Cook et al. [5] performed radiomic analysis with PET/CT and revealed that neighborhood gray-tone difference matrix (NGTDM) contrast at the six-week follow-up, and the percentage change in first-order entropy, were independently associated with survival. In our study, GLCM contrast was included in the top four features, but was not an independent predictor of PFS. In addition, none of the percentage-change features showed statistical significance. The primary difference between our study and the one conducted by Cook et al. [5] is the imaging modality of choice. Future prospective studies involving CT features, PET/CT features, and clinical factors are warranted with large numbers of patients.

We acknowledge a number of limitations to this study. First, we only dealt with a small number of patients. Thus, a separate independent group of patients for testing (or validation) was not available. In addition, k-fold and leave-one-out cross validation were not applied to the study, as such methods yield substantial variability relative to the small sample [34] and were not considered suitable for the current study population. Instead, we performed bootstrapping to calculate the concordance index. Furthermore, the exclusion of the major proporiton of patients was to the absence of contrast enhanced CT scans. This exclusion criteria might have induced selection bias as patients with more complicated disease status requiring detailed evaluation of the mediastinum, vessles, or distant metastatic lesions might be chosen. Second, CT protocols were heterogeneous with multiple scanners and acquisition settings. Radiomic features are influenced by both CT scanners [35] and protocols [20, 24, 36, 37]. Therefore, homogeneous and standardized CT acquisition is necessary. However, Aerts et al. [11] showed that the prognostic performance of radiomic features was reproducible with heterogeneous sets of CT images. In fact, the clinical implications of radiomic feature variability (according to CT scanning) have yet to be determined. The effect of scanning protocols on the actual performance of radiomic features is an issue for future analysis. Third, we dealt with only a fraction of radiomic features. A single GLRL-based feature (grey-level nonuniformity) was calculated based on the study by Aerts et al. [11]. However, filter methods such as Laplacian of Gaussian, or the fractal dimension method, were not tested in the present study. Therefore, our study results cannot be applied to features obtained through those methods.

In conclusion, radiomic features enable PFS estimation in *EGFR*-mutant adenocarcinoma patients treated with first-line EGFR TKIs. Radiomic features combined with clinical factors provided significant improvements in prognostic performance compared to clinical factors alone and may contribute to better therapeutic decision making for individual patients in clinical practice.

## Supporting information

S1 Dataset Radiomic feature data and clinical characteristics of the study population. (XLSX)

S1 Protocol CT scanning protocols for adenocarcinoma patients treated with EGFR TKIs and a CT scanning protocol for a separate group of patients with solid pulmonary nodule to analyze inter-reader intraclass correlation coefficients of radiomic features. (DOCX)
