# Supplementary material for: The prognostic value of CT radiomic features for patients with pulmonary adenocarcinoma treated with EGFR tyrosine kinase inhibitors
Source: PLoS One. 2017 Nov 3;12(11):e0187500. doi: 10.1371/journal.pone.0187500 (PMC5669442; doi:10.1371/journal.pone.0187500)
Supplement: S1 Protocol — (DOCX) [file pone.0187500.s002.docx]

**CT Scanning Protocols for Adenocarcinoma Patients Treated with Epidermal Growth Factor Receptor Tyrosine Kinase Inhibitors**

1. Light Speed Ultra, GE Healthcare, Waukesha, WI

Detailed scanning parameters were as follows: 1.25 x 8 mm detector collimation, 120 kVp, 16.36 noise index with Auto mA, 0.5 sec gantry rotation time, pitch of 1.35, 512 x 512 matrix, mediastinum reconstruction kernel, 2.5 mm reconstruction increment, and section thicknesses of 2.5 mm.

2. Discovery CT750HD, GE Healthcare, Waukesha, WI

Detailed scanning parameters were as follows: 0.625 x 64 mm detector collimation, 120 kVp, 22.67 noise index with Smart mA, 0.5 sec gantry rotation time, pitch of 0.984, 512 x 512 matrix, chest reconstruction kernel, 2.5 mm reconstruction increment, and section thicknesses of 2.5 mm.

3. Sensation 16, Siemens Healthcare, Forchheim, Germany

Detailed scanning parameters were as follows: 0.75 x 16 mm detector collimation, 120 kVp, 100 quality-reference mAs with CARE Dose 4D, 0.5 sec gantry rotation time, pitch of 1, 512 x 512 matrix, B30f reconstruction kernel, 3 mm reconstruction increment, and section thicknesses of 3 mm.

4. Definition, Siemens Healthcare, Forchheim, Germany

Detailed scanning parameters were as follows: 0.6 x 64 mm detector collimation, 120 kVp, 150 quality-reference mAs with CARE Dose 4D, 0.5 sec gantry rotation time, pitch of 1, 512 x 512 matrix, B30f reconstruction kernel, 3 mm reconstruction increment, and section thicknesses of 3 mm.

5. Brilliance 64, Philips Healthcare, Cleveland, OH;

Detailed scanning parameters were as follows: 0.625 x 64 mm detector collimation, 120 kVp, 200 reference mAs with Doseright ACS and Z DOM, 0.5 sec gantry rotation time, pitch of 0.515, 512 x 512 matrix, YC-0.75 reconstruction kernel, 3 mm reconstruction increment, and section thicknesses of 3 mm.

6. Ingenuity, Philips Healthcare, Cleveland, OH

Detailed scanning parameters were as follows: 0.625 x 64 mm detector collimation, 120 kVp, 170 reference mAs with Doseright ACS and Z DOM, 0.5 sec gantry rotation time, pitch of 1.0015, 512 x 512 matrix, YC-0.75 reconstruction kernel, 3 mm reconstruction increment, and section thicknesses of 3 mm.

* All patients were scanned in the supine position from the lung apex to the base during suspended maximum inspiration.

* For contrast enhancement, a total of 70-90mL of 370mgI/mL of the contrast material, iopamidol (Pamiray 370; Dongkook Pharmaceutical, Seoul, Korea) or iopromide (Ultravist 370; Schering, Berlin, Germany), was injected at a rate of 2.3-3.0mL/sec using a power injector. CT scanning was performed with a 60-second delay.

**CT Scanning Protocol for a Separate Group of Patients with Solid Pulmonary Nodule to Analyze Inter-reader Intraclass Correlation Coefficients of Radiomic Features**

1. Definition, Siemens Healthcare, Forchheim, Germany

Detailed scanning parameters were as follows: 0.6 x 64 mm detector collimation, 120 kVp, 150 quality-reference mAs with CARE Dose 4D, 0.5sec gantry rotation time, pitch of 1, 512 x 512 matrix, B50f reconstruction kernel, 1.0mm reconstruction increment, with a section thickness of 1.0mm. A total of 70-90mL of 370mgI/mL of the contrast material, iopromide (Ultravist 370; Schering, Berlin, Germany), was injected at a rate of 2.3-3.0mL/sec using a power injector. The CT scans were initiated 60 seconds after the start of the contrast administration.
